# Sirenomelia or "Mermaid Syndrome" in a Twin Pregnancy: A Case Report

**DOI:** 10.7759/cureus.34311

**Published:** 2023-01-28

**Authors:** Annie F Siddiqui, Vaibhav P Anjankar

**Affiliations:** 1 Anatomy, Jawaharlal Nehru Medical College, Datta Meghe Institute of Medical Sciences, Wardha, IND

**Keywords:** stillborn baby, mermaid baby syndrome, stillbirth, mermaid syndrome, mermaid, rare, fatal, congenital, twin pregnancy, sirenomelia

## Abstract

Sirenomelia, also known as "mermaid syndrome" or "mermaid baby syndrome," is a very rare congenital disorder. The major anomaly in this syndrome is the fusion of the lower legs, giving it a mermaid-like appearance. This syndrome consists of a range of abnormalities affecting various systems, such as the digestive, genitourinary, and musculoskeletal systems. On the basis of the severity of the syndrome, the fetus may have a single fused bone or entirely absent bones in place of a normal pair of distinct bones. In major cases, mermaid syndrome leads to stillbirths. Its occurrence in monozygotic twins is much greater than in dizygotic twins or in a single fetus. The syndrome is believed to mainly occur in cases of maternal age less than 20 years or more than 40 years, women suffering from maternal diabetes, and prenatal exposure to retinoic acid, cocaine, and water contaminated by landfills.

A 22-year-old pregnant female was admitted with a history of amenorrhea for nine months (full-term twin pregnancy) and oligohydramnios for a caesarian section. This was the patient's second pregnancy. A cesarean section was done as instructed by the gynecologist. The patient delivered twin babies. In this twin pregnancy, the first baby was normal and healthy, while the second baby was stillborn and suffered from mermaid syndrome.

## Introduction

Sirenomelia, otherwise known as "mermaid syndrome," is a very rare congenital anomaly in which the legs are "fused together," giving them the resemblance of a mermaid’s tail. It has the same name as the legendary Greek sirens. There are some anomalies, such as anorectal and genitourinary defects, associated with this condition. In normal pregnancies, this condition occurs between 0.1 and 0.025 times per 10,000. Up to 22% of fetuses with this defect will have mothers who have pregestational diabetes without proper glucose adjustment, and there is a substantial correlation between this syndrome and maternal diabetes, with a comparative risk of 1:200-250 [1].

We describe a case of mermaid syndrome that was discovered at birth. The primary anatomical characteristic separating sirenomelia from caudal regression syndrome is the existence of a single umbilical artery originating from the vitelline artery. The safest medical option to date is to diagnose this problem in the first trimester and seek counseling and advice for the termination of pregnancy. The greater part of the instances of sirenomelia bring about stillbirth, and this condition is multiple times higher in monochorionic twins than in single-birth or dichorionic twins. The condition is caused by the lack of mesodermal movement in the caudal locale of the undeveloped organism during growth. The mesoderm of this locale is engaged with the advancement of the lumbosacral vertebra and the urogenital and gastrointestinal systems. Among other related birth deformities, the newborn child with this condition is, in many cases, brought into the world with renal agenesis, abnormalities of the urogenital system, including the urethra, and abnormalities in the umbilical cord insertion [1,2].

In the perinatal stage, it is a fatal condition. Due to several congenital abnormalities, sirenomelia live births have meager survival and poor quality of life [2]. The incidence of sirenomelia in monozygotic twins is 100 times higher than that of singleton pregnancy and dizygotic twins [3].

## Case presentation

A 28-year-old woman, gravida two, para one, with one living, healthy female child of four years, was admitted for a cesarean section for an indication of 38 + 4 weeks of dichorionic and diamniotic twin pregnancy with oligohydramnios. Her old antenatal USG showed an intrauterine twin pregnancy with a dichorionic and diamniotic twin pregnancy and severe dilation of the frontal and occipital horns of the lateral ventricle of the second fetus. The first baby was normal and healthy. A repeat antenatal ultrasound done at the time of admission showed twin live fetuses; the first baby was 35 weeks +/- three weeks and the presentation was cephalic with a weight of 2451 grams and a fetal heart rate (FHR) of 143 beats per minute. The second baby had a breech presentation and was 31 weeks +/- three weeks old, with no heart rate found. The weight of this fetus was found to be 1800 grams. Holoprosencephaly was noted in the second fetus.

On examination of the patient, her blood pressure was 110/80 mm Hg and her pulse was 74/min. Pallor and mild edema were seen in the patient. On abdominal examination, it was found to be distended, and multiple fetal parts were felt. Twin A was in cephalic presentation and had a regular fetal heart sound. On vaginal examination, the OS was closed, there was no show, and no leaking was observed.

**Figure 1:**
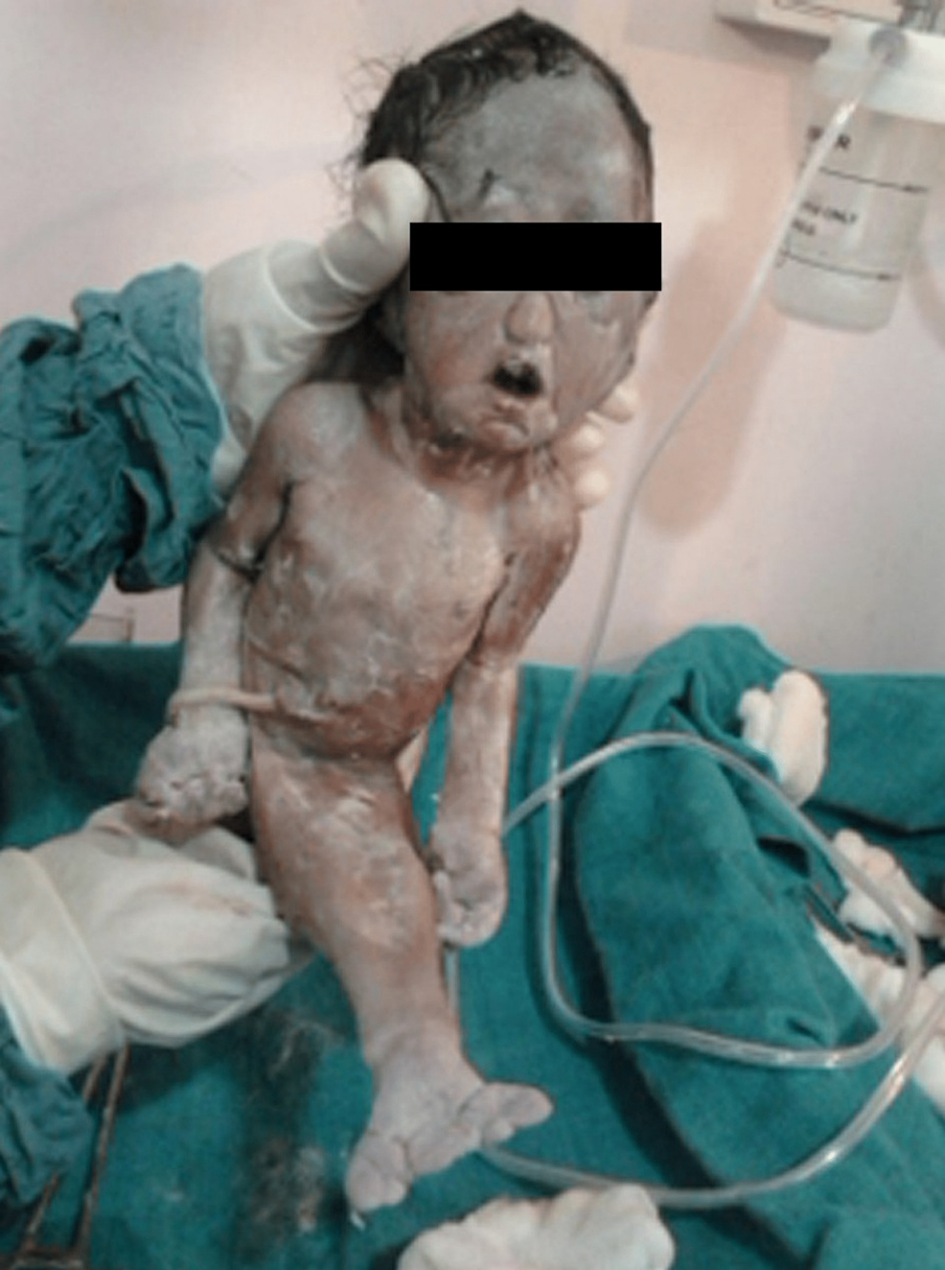
The second fetus showing mermaid syndrome with fused lower limbs, absent external genitalia, and anus.

**Figure 2:**
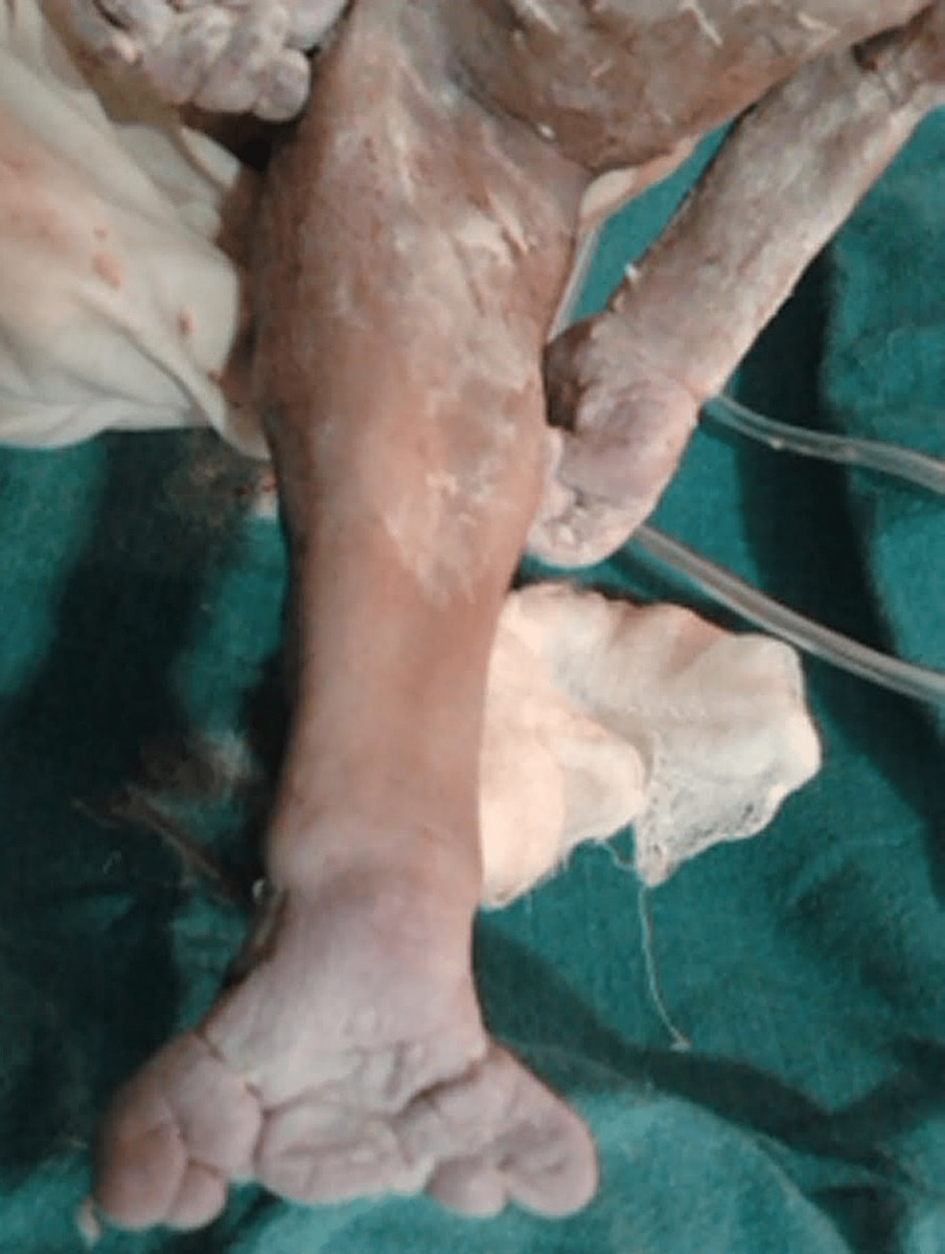
Fused lower limbs giving the appearance of a mermaid’s tail.

Preoperative work was completed. Then a cesarean section was performed under spinal anesthesia. The first baby, delivered by the head, weighed 2.5 kg. This baby was found to be crying immediately after birth and was handed over to a pediatrician. The second baby was delivered breech with a congenital anomaly known as sirenomelia (Figure 1). That is the bilaterally fused lower extremities with absent external genitalia and anus show mermaid baby syndrome (Figure 2).

The second baby died immediately after birth. Parents refused to do further post-mortem investigations like an autopsy and a radiological examination; hence, the baby was handed over to the relatives. The mother and normal (first) baby were discharged on the fifth day of the caesarian section.

## Discussion

Sirenomelia, also known as mermaid syndrome and best defined by Stevenson as "a limb anomaly in which the normally paired lower limbs are replaced by a single midline limb," is a rare congenital malformation disorder with a prevalence of approximately one per 100,000. A range of abnormalities mainly affect the musculoskeletal, genitourinary, and digestive systems. Depending on the severity of the condition, the deformity may present as single fused bones where there usually are pairs of two distinct bones or entirely absent bones [4]. According to the reports, sirenomelia is more common in one of every two monozygotic twins [5]. Sirenomelia, a fatal congenital disorder affecting the embryonic body's caudal region, is present at birth. However, the fusion of the lower limbs is the most noticeable characteristic. According to estimates, 49.5% of pregnancies end willingly because of fetal abnormalities. The exact cause of the anomaly is still unknown; however, it is thought to be caused by a combination of genes and environmental factors [6].

The precise cause of sirenomelia is unknown. The probable reasons are pregnancies in women younger than 20 or older than 40; maternal diabetes; prenatal exposure to retinoic acid, cocaine, and water contaminated by landfills, etc. [7].

An adequate classification of Sirenomelia is reported by Stocker and Heifetz. I: all thigh and leg bones are present; II: single fibula; III: absent fibulae; IV: partially fused femurs, fused fibulae; V: partially fused femurs, absent fibulae; VI: single femur, single tibia; VII: single femur, absent tibiae. In this case, the sirenomelia twin was classified as type III according to ultrasonography and clinical examination, with fused lower extremities and absent fibula and tibia [8].

The two most likely theories are the vascular steel theory and faulty blastogenesis. According to the vascular steel theory, the abnormal abdominal artery, which sends a lot of blood to the placenta, develops from the high level of the aorta without any branches from the iliac or renal arteries. The inferior region of the body will develop abnormally or incorrectly due to improper caudal circulation. Defective blastogenesis: between the 13th and 22nd day, it may cause the fusion of the lower part of the body, malrotation, or dysgenesis [9].

To look for and identify this congenital defect early, it is advised to have routine prenatal ultrasound examinations. A technique for improving complete anatomical visualization is the transvaginal scan, although imaging of the sirenomelia by ultrasound may be hampered by synchronized oligohydramnios and intrauterine growth retardation. Ultrasonography is an appropriate method for detecting sirenomelia in the first or early second trimester (intrauterine growth restriction) [10]. Magnetic resonance imaging (MRI) and three-dimensional sonography are also crucial in diagnosing sirenomelia. To confirm the diagnosis, a postnatal X-ray and autopsy are also recommended [11].

## Conclusions

Sirenomelia is a very rare and fatal congenital abnormality. It can be diagnosed via imaging in an antenatal ultrasound examination or post-natal radiography of the lower limb bones of the baby. Antenatal diagnosis by ultrasonography in the first and second trimesters is essential for the early diagnosis and management of sirenomelia.
